# Subsequent Meningiomas Among Survivors of Childhood Cancer

**DOI:** 10.1001/jamanetworkopen.2025.48715

**Published:** 2025-12-11

**Authors:** Daniel C. Bowers, Table Cooney, Yan Chen, Yan Yuan, Robert Galvin, Cindy Im, Wendy Leisenring, Samuel W. Brady, Susan A. Smith, Rebecca M. Howell, Michael A. Arnold, Miriam Conces, Yutaka Yasui, Lisa R. Diller, Gregory T. Armstrong, Joseph P. Neglia, Lucie M. Turcotte

**Affiliations:** 1 Division of Pediatric Hematology/Oncology, Simmons Comprehensive Cancer Center, University of Texas Southwestern Medical School, Dallas; 2 Dana Farber/Boston Children’s Cancer and Blood Disorders Center, Harvard Medical School, Boston, Massachusetts; 3 Tango Therapeutics, Boston, Massachusetts; 4 St Jude Children’s Research Hospital, Memphis, Tennessee; 5 University of Alberta, Edmonton, Alberta, Canada; 6 University of Minnesota, Minneapolis; 7 Fred Hutchinson Cancer Research Center, Seattle, Washington; 8 The University of Texas MD Anderson Cancer Center, Houston; 9 Children’s Hospital Colorado and the University of Colorado, Anschutz Medical Campus, Aurora; 10 Nationwide Children’s, Columbus, Ohio

## Abstract

**Importance:**

Associations with chemotherapy, occurrence of multiple meningiomas, and mortality after subsequent meningioma diagnosis among survivors of childhood cancer remain unclear.

**Objectives:**

To report the incidence of meningioma among childhood cancer survivors, identify novel risk factors for meningiomas, characterize survivors with multiple meningiomas, and describe cause-specific mortality following meningioma occurrence.

**Design, Setting, and Participants:**

The Childhood Cancer Survivor Study is a retrospective cohort study with longitudinal prospective follow-up of childhood cancer survivors diagnosed between 1970 to 1999 in the US and Canada. Eligibility included diagnosis of cancer before age 21 years and surviving more than 5 years after diagnosis. Meningiomas were self-reported and confirmed by review of pathology reports. Childhood cancer diagnosis, chemotherapy details, and radiation therapy exposures from up to 5 years from diagnosis were abstracted from medical records.

**Main Outcomes and Measures:**

Cumulative incidence of meningioma was calculated starting from 5 years from the diagnosis. Overall survival (OS) from diagnosis of the first subsequent meningioma was estimated using Kaplan-Meier methods.

**Results:**

The CCSS cohort included 24 886 survivors initially diagnosed from 1970 to 1999, including 471 survivors (263 female [56%]; median [range] age at last follow-up, 42.5 [19.7-66.3] years; median [range] age at primary cancer diagnosis, 5.6 [0-20.9] years) who were diagnosed with 710 meningiomas. Thirty-five years after primary cancer diagnosis, the cumulative incidence of a subsequent meningioma was 2.3% (95% CI, 2.1%-2.6%). Of the 471 survivors who developed meningioma, 137 (29.0%) had at least 2 meningiomas, and 80 (16%) met criteria for meningiomatosis. An increased risk of meningioma was associated with higher doses of cranial radiation therapy (eg, HR, 125.3 [95% CI, 58.1-270.5]), younger age at primary cancer diagnosis (eg, 0 to 4 years: HR, 4.0 [95% CI, 2.4-6.1]), female sex (HR, 1.6 [95% CI, 1.3-1.9]), and exposure to platinum, antimetabolite chemotherapy, and intrathecal chemotherapy, and a lower risk was associated with non-Hispanic Black race (HR, 0.5 [95% CI, 0.3-1.0]) and exposure to alkylating agents (HR, 0.6 [95% CI, 0.5-0.8]). The all-cause cumulative mortality was 4.9%, 10.5% and 18.4% at 5, 10, and 15 years from the first subsequent meningioma diagnosis.

**Conclusions and Relevance:**

Meningiomas have a relatively high incidence and mortality for childhood cancer survivors. Results from this study could justify screening for meningiomas in high-risk populations.

## Key Points

**Question:**

What are the incidence, risk factors, and mortality for single and multiple meningiomas among survivors of childhood cancer?

**Findings:**

In this cohort study of 24 886 survivors in the Childhood Cancer Survivor Study cohort, 471 survivors were diagnosed with 710 meningiomas; 35 after primary cancer diagnosis, the cumulative incidence of a subsequent meningioma was 2.3%. Meningioma risk was associated with high doses of radiation therapy, young age at primary cancer diagnosis, female sex, and exposure to platinum, antimetabolite chemotherapy, and intrathecal chemotherapy, and high rates of all-cause mortality were observed following meningioma diagnosis.

**Meaning:**

These findings suggest that certain high-risk populations of childhood cancer survivors may be suitable candidates for screening for subsequent meningiomas.

## Teaser

This cohort study examines the incidence, risk factors, and mortality of meningioma among childhood cancer survivors using data from the Childhood Cancer Survivor Study.

## Introduction

Currently, 85% of children diagnosed with cancer will survive for 5 years or more, leading to an estimated 580 000 survivors of childhood cancer in the US by 2040.^1^ However, these survivors face significantly higher risks of morbidity and early mortality than the general population.^2,3^ Meningiomas are the most common subsequent central nervous system (CNS) tumor among aging adult survivors of childhood cancer, yet relatively few studies report their incidence, risk factors, and outcomes.^4,5,6,7,8,9,10^ And because meningiomas are often indolent in their presentation and histologically benign, clinical treatment decisions between aggressive surgical approaches and observation are challenging.^11^

Exposure to cranial radiation therapy (CRT) for primary CNS malignancies and head and neck cancers has long been recognized as a significant risk factor for the development of meningiomas.^5,6,12,13,14,15,16,17,18,19^ In addition to CRT exposure, other risk factors include cancer-type diagnosis and younger ages at the time of diagnosis and treatment.^7,8,19^ Exposure to chemotherapy agents, including methotrexate^7,12^ and carboplatin,^8^ has been occasionally associated with subsequent meningiomas; however, these risk factors have not been consistently identified across studies.

The cause-specific mortality of subsequent meningiomas has not been well characterized.^4,12,20,21^ In addition, while multiple meningiomas have been described in childhood cancer survivors,^12^ these are poorly described as they are not distinguished between recurrence of the original tumor vs distinctly new subsequent tumors.

The primary objectives of this study were to use the Childhood Cancer Survivor Study (CCSS) cohort to report the incidence of meningioma across the lifespan, to identify novel risk factors for meningiomas, to characterize survivors with multiple meningiomas, and to describe cause-specific mortality following meningioma occurrence.

## Methods

### Study Population

The Childhood Cancer Survivor Study (CCSS) is a retrospectively constructed cohort study with longitudinal prospective follow-up of survivors of childhood cancer treated at 31 institutions in the US and Canada. Eligibility for participation in the CCSS included diagnosis of cancer before age 21 years between 1970 and 1999, and surviving at least 5 years after diagnosis of leukemia, CNS tumor, Hodgkin or non-Hodgkin lymphoma, Wilms tumor, neuroblastoma, soft tissue sarcoma, or bone tumor. The cohort methods and study design have been described previously.^22,23^ The CCSS was approved by institutional review boards at the participating centers, and participants provided informed consent. This study was conducted in accordance with the Strengthening the Reporting of Observational Studies in Epidemiology (STROBE) reporting guideline.

### Childhood Cancer Treatment Information and Meningioma Identification

Childhood cancer diagnosis details and therapeutic exposures from up to 5 years from diagnosis were abstracted using a systematic protocol from medical records obtained from treating institutions. Chemotherapy exposures were identified for both individual agents and classes of agents with routes of administration, summarized as both yes or no and cumulative dose. For survivors treated with radiation therapy (RT), detailed radiation records were collected and uniformly abstracted at the CCSS Radiation Physics Center for specific parameters, including target dose delivered.^24,25^

Meningiomas were identified initially through self- or proxy report. They were confirmed by review of pathology reports if available or alternatively by other medical records, including radiology reports, or by death certificates. Survivors with multiple meningiomas and meningiomatosis were included in the analysis. Meningiomatosis was defined by *International Statistical Classification of Diseases and Related Health Problems for Oncology* code 9530/1 or by record review of multiple meningiomas on the same diagnostic date.^26^

Vital status, death date, and cause of death were primarily ascertained by National Death Index record review (completed December 31, 2017). Causes of death were adjudicated with standardized rules based on the *International Classification of Diseases, Ninth Revision *and *Tenth Revision*.

### Statistical Analysis

Survivors of childhood cancer were considered at risk of meningiomas beginning at 5 years after their childhood cancer diagnosis, until a confirmed diagnosis of meningioma, death, or date of most recent contact. Cumulative incidence of meningioma overall, by sex, by treatment exposure, by treatment era, and by age at diagnosis were calculated with a nonparametric estimate using time from childhood cancer diagnosis as the time scale, starting from 5 years from the diagnosis, and treating death as a competing risk event; those without events and were alive at last follow-up were censored.^27,28^ Overall survival (OS) from diagnosis of the first subsequent meningioma was estimated using Kaplan-Meier methods.

Cox regression estimated the relative hazards of a first meningioma diagnosis treating death as a censoring event. Using age as the timescale, where the time origin was set at 5 years after diagnosis and time at risk ended at the first event, last follow-up, or death, models assessed variables that modify the risk of a first meningioma diagnosis. Univariate analyses were used to evaluate the effect of era of diagnosis, sex, race and ethnicity, age at diagnosis, primary cancer diagnosis, CRT exposure (dichotomous variable) and radiation dose, chemotherapy exposure (including alkylating agents, antimetabolites, intrathecal methotrexate, platinum agents, epipodophyllotoxins, and anthracycline), and hematopoietic cell transplantation, adjusting for sex, race, and age at diagnosis. The cyclophosphamide equivalent dose (CED)^29^ was used to calculate alkylating agent dose; antimetabolites included methotrexate, 6-mercaptopurine, 6-thioguanine, and cytarabine. All variables with a *P* value smaller than .10 were then entered in a multivariable model, adjusting for sex, race and ethnicity, and age at diagnosis. Because cancer diagnosis and cancer treatments are highly correlated, these were separated into 2 models.

Analyses were completed using SAS version 9.4 (SAS Institute).^30^ Descriptive statistics and model results accounted for sampling weights reflecting the CCSS undersampling of acute lymphoblastic leukemia survivors by study design. Two-sided *P* < .05 was considered statistically significant. Data were analyzed from June 2022 to August 2024.

## Results

**Figure 1.**
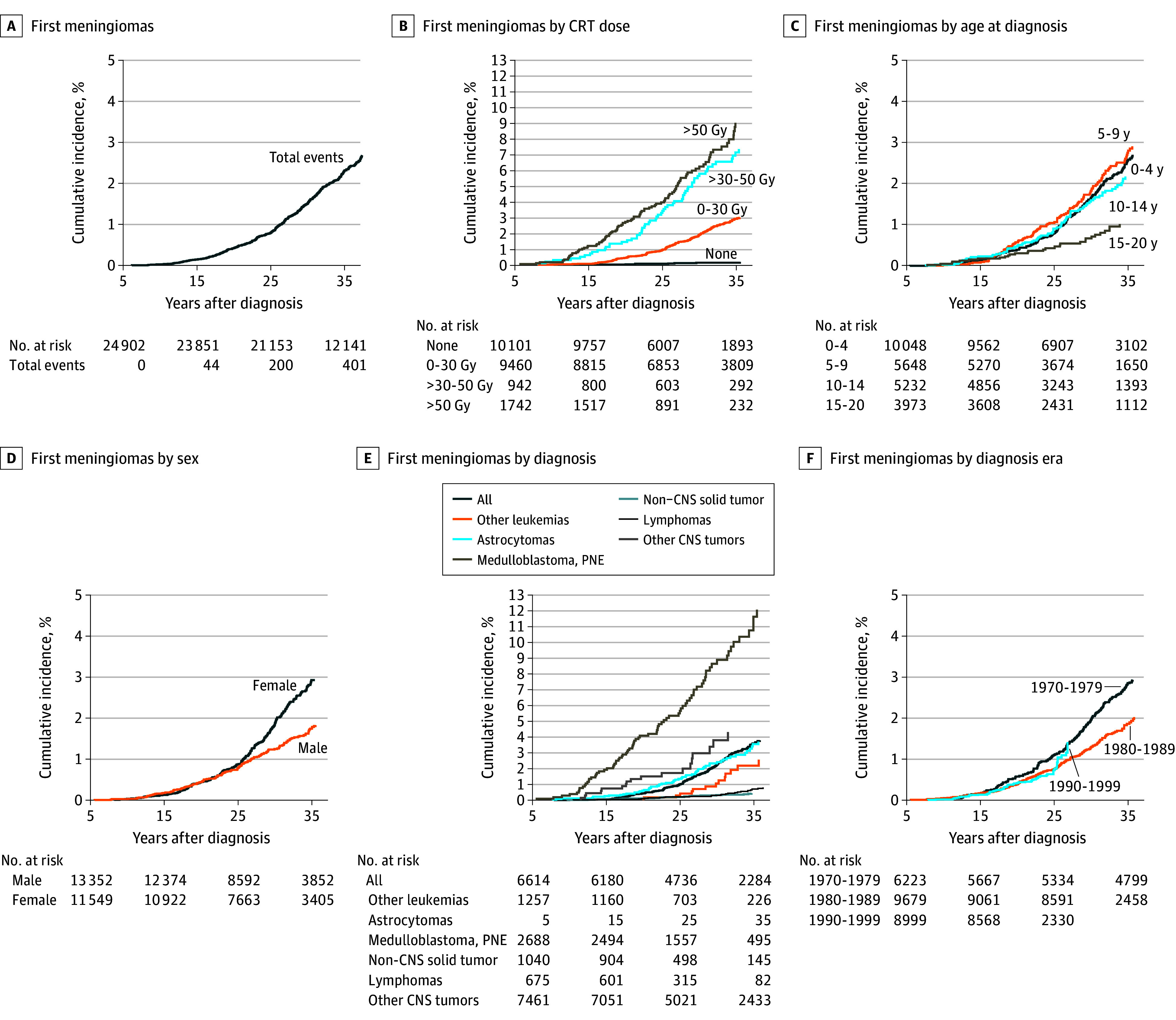
Cumulative Incidence of Subsequent Meningiomas for All Survivors, Dose of Cranial Radiation, Age at Primary Cancer Diagnosis, Female vs Male, Primary Cancer Diagnosis, and Diagnostic Era CNS indicates central nervous system; CRT, cranial radiation therapy.

Among the 24 886 survivors in the CCSS cohort, 471 survivors were diagnosed with 710 meningiomas (Table 1). Thirty-five years after primary cancer diagnosis, the cumulative incidence of a subsequent meningioma was 2.3% (95% CI, 2.1%-2.6%) (Figure 1A). Among the 471 survivors with a subsequent meningioma, the median (range) age at last follow-up was 42.5 (19.7-66.3) years. Median (range) age at primary cancer diagnosis was 5.6 (0-20.9) years and 263 (56%) were female. Among survivors diagnosed with meningioma, the median (range) age at diagnosis of their first meningioma was 32.3 (7.6-58.5) years, the median (range) interval from primary cancer diagnosis to first meningioma diagnosis was 26.2 (5.6-48.2) years.

**Table 1.** Clinical Characteristics of Childhood Cancer Survivors Who Did and Did Not Develop Meningiomas^**a**^

Characteristic	Patient, No. (%)				*P* value^b^
	Any meningioma	≥2 Meningioma	Meningiomatosis^c^	No meningioma	
No.	471	137	80	24 415	
Age at diagnosis of primary cancer, median (range), y	5.6 (0-20.9)	5.3 (1.0-20.9)	5.1 (1.1-17.2)	6.0 (0-21)	<.001
Age at primary cancer diagnosis, y					
0-4	214 (45.1)	60 (44.7)	39 (48.8)	9831 (43.3)	<.001
5-9	136 (28.8)	47 (33.6)	26 (32.5)	5507 (23.8)	
10-14	88 (19.3)	23 (16.6)	14 (17.5)	5138 (18.9)	
15-20	33 (6.7)	7 (5.1)	1 (1.3)	3939 (14.0)	
Age at last follow-up, median (range), y	42.5 (19.7-66.3)	45.6 (23.8-66.3)	46.0 (23.8-62.5)	35.7 (5.6-68.1)	<.001
Ages at last follow-up, mean (SD), y					
<20	1 (0.2)	0	0	1100 (4.3)	<.001
20-29	37 (8.0)	7 (5.0)	3 (3.8)	5030 (25.4)	
30-39	131 (30.7)	31 (24.3)	16 (20.0)	8490 (35.5)	
40-39	205 (41.7)	66 (47.1)	44 (55.0)	6799 (24.2)	
≥50	97 (19.4)	33 (23.6)	17 (21.3)	2996 (10.6)	
Sex					
Male	208 (44.0)	57 (42.9)	29 (36.3)	13 133 (53.8)	<.001
Female	263 (56.0)	80 (57.1)	51 (63.8)	11 282 (46.2)	
Race and ethnicity					
Hispanic/Latine	32 (7.5)	7 (5.0)	6 (7.5)	1967 (8.8)	<.001
Non-Hispanic Black	12 (2.4)	3 (2.1)	3 (3.8)	1592 (6.6)	
Non-Hispanic White	408 (85.7)	122 (87.4)	71 (88.8)	19 828 (80.2)	
Other race/unknown	19 (4.4)	5 (5.5)	0 (0.0)	1028 (4.4)	
Primary cancer diagnosis					
Non-CNS solid tumor	27 (5.4)	4 (2.9)	2 (2.5)	7433 (26.2)	<.001
Acute lymphoblastic leukemia	232 (52.1)	74 (55.0)	43 (53.8)	6383 (36.5)	
Other leukemias	15 (3.0)	3 (2.1)	2 (2.5)	1242 (4.3)	
Astrocytoma	66 (13.2)	22 (15.7)	12 (15.0)	2614 (9.2)	
Medulloblastoma	84 (16.8)	26 (18.6)	16 (20.0)	952 (3.4)	
Other CNS tumors	18 (3.6)	3 (2.1)	2 (2.5)	654 (2.3)	
Lymphomas	29 (5.8)	5 (3.6)	3 (3.8)	5137 (18.1)	
Decade of diagnosis of primary cancer					
1970-1979	241 (48.3)	81 (57.8)	52 (65.0)	5982 (21.1)	<.001
1980-1989	168 (35.3)	43 (30.7)	22 (27.5)	9508 (36.6)	
1990-1999	62 (16.4)	13 (11.5)	6 (7.5)	8925 (42.4)	
Treatment for primary childhood cancer					
Surgery only	5 (1.1)	1 (0.8)	1 (1.3)	1950 (7.5)	<.001
Radiotherapy only	1 (0.2)	1 (0.8)	0 (0.0)	62 (0.2)	
Chemotherapy only	3 (1.2)	0 (0.0)	0 (0.0)	2624 (18.9)	
Surgery and radiotherapy	82 (17.7)	22 (17.1)	14 (18.4)	1816 (7.0)	
Surgery and chemotherapy	6 (1.3)	0 (0.0)	0 (0.0)	5415 (23.3)	
Chemotherapy and radiotherapy	156 (37.9)	47 (38.7)	29 (38.2)	2183 (10.8)	
Surgery, chemotherapy, and radiotherapy	182 (40.5)	55 (42.7)	32 (42.1)	8097 (32.3)	
Cranial radiation dose, Gy					
None	14 (3.7)	1 (0.8)	1 (1.4)	10 088 (50.9)	<.001
>0-30.0	249 (59.6)	40 (55.6)	71 (58.2)	9211 (39.3)	
30.1-50.0	65 (14.1)	13 (18.1)	22 (17.3)	877 (3.5)	
>50.0	104 (22.6)	18 (25.0)	30 (23.7)	1622 (6.3)	
Age at first meningioma, median (range), y	32.3 (7.6-58.5)	33.3 (7.6-55.1)	35.6 (13.1-55.1)	NA	.014
Age at first meningioma, y					
5-19	34 (6.9)	14 (10.0)	7 (8.8)	NA	NA
20-39	323 (70.3)	85 (62.9)	48 (60.0)	NA	
40-49	97 (19.5)	31 (22.1)	20 (25.0)	NA	
≥50	17 (3.4)	7 (5.0)	5 (6.3)	NA	
Alkylating agents (CED), mg/m^2^					
None	235 (56.7)	74 (63.4)	47 (65.3)	10 045 (47.5)	<.001
0 to <4000	40 (9.2)	10 (8.3)	6 (8.3)	2773 (16.5)	
≥4000 to <8000	34 (9.8)	8 (6.6)	5 (6.9)	2754 (12.6)	
≥8000	102 (24.3)	26 (21.7)	14 (19.4)	5178 (23.4)	
Antimetabolite agent exposure					
Yes	254 (61.7)	77 (62.9)	47 (62.7)	10 077 (53.4)	<.001
No	175 (38.3)	47 (37.1)	28 (37.3)	12 163 (46.6)	
Intrathecal methotrexate exposure					
Yes	213 (53.5)	70 (58.8)	41 (56.9)	7273 (42.7)	<.001
No	209 (46.5)	51 (41.2)	31 (43.1)	14 861 (57.3)	
Platinum agent exposure, mg/m^2^					
None	381 (88.9)	113 (91.3)	68 (90.7)	19 740 (90.8)	.09
0 to <400	26 (5.7)	6 (4.7)	4 (5.3)	925 (3.6)	
≥400 to <750	19 (4.1)	4 (3.2)	2 (2.7)	992 (3.8)	
≥750	6 (1.3)	1 (0.8)	1 (1.3)	478 (1.8)	
Years between the first and the second meningiomas median (range), y	NA	1.4 (0-30.2)	NA	NA	NA

Abbreviations: CED, cyclophosphamide equivalent dose; CNS, central nervous system; NA, not applicable.

^a^ Except the count, all other numbers considered the sample weight. The percentages considered the sampling weight and are based on the n total number of participants whom information was available.

^b^ *P* value compares any meningioma vs no meningioma.

^c^ Meningiomatosis is defined by the *International Statistical Classification of Diseases and Related Health Problems for Oncology* diagnosis code 9530.1 or multiple meningiomas diagnosed the same date.

Additionally, 137 of 471 survivors (29%) with meningiomas had more than 1 meningioma, and 80 (17%) met predefined criteria for meningiomatosis. For survivors with at least 2 subsequent meningiomas (including those with meningiomatosis), the median (range) time interval between the first and second meningiomas was 1.4 (0-30.2) years. Among survivors with a meningioma, 14 survivors (3.7%) were not exposed to CRT; 249 survivors (59.6%) were exposed to 0.1-30.0 Gy; 65 survivors (14.1%) were exposed to more than 30.0 to 50.0 Gy; and 104 survivors (22.6%) were exposed to more than 50.0 Gy (Table 1).

### Risk Factor Analysis for Subsequent Meningioma

The cumulative incidence of meningioma for survivors exposed to CRT increased in a dose-dependent manner (Figure 1B). At 35 years following a cancer diagnosis, the cumulative incidence of subsequent meningioma for individuals not exposed to CRT was 0.14% (95% CI, 0.08%-0.25%). In contrast, for those exposed to CRT doses of 0 to 29.0 Gy, 30.0 to 49.0 Gy, and more than 50.0 Gy, the cumulative incidences were 2.9% (95% CI, 2.6%-3.3%), 7.1% (95% CI, 5.5%-9.1%), and 9.0% (95% CI, 7.2%-11.1%), respectively. The cumulative incidence of subsequent meningioma was higher in children compared with young adults (Figure 1C). Among survivors aged 15 to 20 years, the cumulative incidence was 1.0% (95% CI, 0.7%-1.5%). For those diagnosed with cancer at ages 0 to 4 years, 5 to 9 years, and 10 to 14 years, the cumulative incidences were 2.6% (95% CI, 2.2%-3.0%), 2.8% (95% CI, 2.3%-3.4%), and 2.2% (95% CI, 1.7%-2.7%), respectively. Additionally, the cumulative incidence of subsequent meningioma was higher in females (2.9%; 95% CI, 2.6%-3.3%) compared with males (1.8%; 95% CI, 1.5%-2.1%) (Figure 1D). Finally, for survivors of medulloblastoma, astrocytoma, and acute lymphoblastic leukemia, the cumulative incidences of subsequent meningiomas were 11.6% (95% CI, 9.0%-14.6%), 4.3% (95% CI, 2.5%-6.8%), and 3.6% (95% CI, 3.1%-4.1%), respectively (Figure 1E).

In multivariable analysis, several risk factors for meningioma were identified (Table 2). These included cumulative CRT dose, with higher doses associated with greater risk compared with no radiation exposure: 1 to 29 Gy (HR, 18.0 [95% CI, 8.5-38.2]), 30 to 49 Gy (HR, 76.5 [95% CI, 34.7-168.4]), and more than 50 Gy (HR, 125.3 [95% CI, 58.1-270.5]). Younger age at primary cancer diagnosis was associated with increased risk of meningioma compared with ages 15 to 20 years (0 to 4 years: HR, 4.0 [95% CI, 2.4-6.1]; 5 to 9 years: HR, 2.7 [95% CI, 1.7-4.2]; 10 to 14 years: HR, 1.86 [95% CI, 1.2-2.9]). Female sex was associated with a higher risk (HR, 1.6 [95% CI, 1.3-1.9]). Non-Hispanic Black survivors had a decreased risk of subsequent meningioma (HR, 0.5 [95% CI, 0.3-1.0]). Chemotherapy exposures were also linked to an increased risk of subsequent meningiomas, including platinum agents (HR, 2.2 [95% CI, 1.5-3.2]), antimetabolite chemotherapy (HR, 1.6 [95% CI, 1.0-2.6]), and intrathecal methotrexate (HR, 2.6 [95% CI, 1.6-4.2]). Exposure to alkylating agents was associated with a reduced risk of subsequent meningiomas (HR, 0.6 [95% CI, 0.5-0.8]; *P* < .001).

**Table 2.** Risk Factors for Subsequent Meningiomas Among Childhood Cancer Survivors

Characteristic, name, level	Univariate		Multivariable analysis^a^			
	HR (95% CI)	Global *P* value	Cancer diagnosis model		Treatment model	
			HR (95% CI)	*P* value	HR (95% CI)	*P* value
Diagnosis era						
1970-1979	1 [Reference]	NA	1 [Reference]	NA	NA	NA
1980-1989	0.61 (0.50-0.76)	<.001	0.73 (0.56-0.95)	.017	0.58 (0.46-0.72)	<.001
1990-1999	0.52 (0.37-0.73)		0.86 (0.51-1.44)	.56	0.51 (0.35-0.74)	<.001
Sex						
Female	1.45 (1.20-1.76)	<.001	1.56 (1.26-1.92)	<.001	1.48 (1.22-1.80)	<.001
Malw	1 [Reference]	NA	1 [Reference]	NA	NA	NA
Race						
Hispanic/Latine	1.06 (0.70-1.59)	.04	1.28 (0.80-2.05)	.30	1.09 (0.72-1.64)	.68
Non-Hispanic Black	0.43 (0.24-0.77)		0.51 (0.26-0.98)	.043	0.51 (0.29-0.92)	.02
Other	1.14 (0.68-1.91)		1.16 (0.63-2.15)	.63	1.16 (0.69-1.94)	.57
Non-Hispanic White	1 [Reference]		1 [Reference]	NA	1 [Reference]	NA
Age at diagnosis						
0-4	6.10 (4.16-8.94)	<.001	3.97 (2.60-6.06)	<.001	3.25 (2.16-4.90)	<.001
5-9	4.93 (3.32-7.32)		2.69 (1.73-4.17)	<.001	2.31 (1.51-3.52)	<.001
10-14	2.77 (1.84-4.19)		1.86 (1.18-2.93)	.008	1.76 (1.15-2.70)	.009
15-20	1 [Reference]	NA	1 [Reference]	NA	NA	NA
Diagnosis						
Acute lymphoblastic leukemia	8.37 (5.58-12.55)	<.001	NA	NA	8.21 (5.46-12.34)	<.001
Other leukemias	3.72 (1.98-7.00)		NA		4.55 (2.40-8.61)	<.001
Astrocytoma	7.50 (4.79-11.75)		NA		8.68 (5.50-13.70)	<.001
Medulloblastoma, PNET	30.92 (19.95-47.91)		NA		36.74 (23.38-57.74)	<.001
Other CNS tumors	9.70 (5.32-17.68)		NA		12.88 (6.95-23.85)	<.001
Lymphomas	1.08 (0.64-1.82)		NA		1.63 (0.94-2.82)	.082
Non-CNS solid tumor	1 [Reference]	NA	NA	NA	NA	NA
Cranial radiation dose						
>0-30 Gy	13.86 (7.49-25.62)	<.001	17.96 (8.46-38.15)	<.001	NA	NA
30.1-50 Gy	43.26 (22.67-82.58)		76.50 (34.74-168.43)	<.001	NA	NA
> 50 Gy	44.92 (23.92-84.38)		125.33 (58.06-270.53)	<.001	NA	NA
None	1 [Reference]	NA	1 [Reference]	NA	NA	NA
Alkylating agent exposure						
Yes	0.74 (0.61-0.91)	.004	0.64 (0.50-0.81)	<.001	NA	NA
No	1 [Reference]	NA	1 [Reference]	NA	NA	NA
Platinum agent exposure						
No	1 [Reference]	NA	1 [Reference]	NA	NA	NA
Yes	1.87 (1.41-2.48)	<.001	2.19 (1.45-3.32)	<.001	NA	NA
Antimetabolites exposure						
Yes	1.60 (1.31-1.96)	<.001	1.61 (1.02-2.56)	.042	NA	NA
No	1 [Reference]	NA	1 [Reference]	NA	NA	NA
Intrathecal methotrexate exposure						
Yes	1.93 (1.57-2.38)	<.001	2.60 (1.61-4.19)	<.001	NA	NA
No	1 [Reference]	NA	1 [Reference]	NA	NA	NA
Systemic methotrexate exposure						
Yes	0.97 (0.75-1.26)	NA	NA	NA	NA	NA
No	1 [Reference]	NA	NA	NA	NA	NA
Hematopoietic cell transplantation						
Yes	1.66 (1.03-2.66)	NA	1.00 (0.58-1.71)	.99	NA	NA
No	1 [Reference]	NA	1 [Reference]	NA	NA	NA

Abbreviations: CNS, central nervous system; HR, hazard ratio; NA, not applicable; PNET, primitive neuroectodermal tumor.

^a^ *P* ≤ .10 in univariate analysis (sex and age at diagnosis are forcing in).

By diagnostic era, the cumulative incidence at 20 years was 0.58% (95% CI, 0.41%-0.79%) for 1970 to 1979, 0.39% (95% CI, 0.28%-0.52%) for 1980 to 1989, and 0.42% (95% CI, 0.32%-0.55%) for 1990 to 1999 (Figure 1F). When comparing the years 1970 to 1979 with later decades of childhood cancer diagnosis, the cumulative incidence of a subsequent meningioma appeared to decrease over time (eTable 1 in Supplement 1). In the multivariable model, compared with the era between 1970 and 1979, the risk of meningioma decreased for the years 1980 to 1989 (HR, 0.73 [95% CI, 0.56-0.95]; *P* = .02), and the decrease in risk was not statistically significant from 1990 to 1999. In addition, when the analysis was restricted to survivors of childhood brain tumors, the risk of meningioma declined for the 1990 to 1999 group (HR, 0.47 [95% CI, 0.26-0.85]; *P* = .01).

### Mortality for Childhood Cancer Survivors With Subsequent Meningiomas

**Figure 2.**
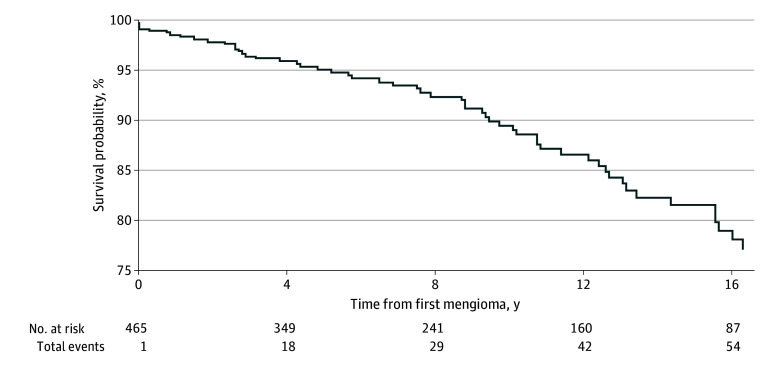
Survival After the Diagnosis of a Meningioma Among 5-Year Survivors of Childhood Cancer

Among the 471 survivors diagnosed with a subsequent meningioma after childhood cancer, the median (range) follow-up after meningioma diagnosis was 8.4 (0-34.5) years and 71 had died (15.1%) (Table 1). The most prevalent cause of death was meningioma (25 [35.2%]) and among those with 2 or more meningiomas, the prevalence of meningioma as the cause of death was higher (7 [53.8%]) (Table 3). The all-cause cumulative mortality was 4.9% (95% CI, 2.9%-7.0%), 10.5% (95% CI, 7.1%-13.9%) and 18.4% (95% CI, 13.3%-23.4%) at 5, 10, and 15 years from the first subsequent meningioma diagnosis, respectively (Figure 2 and eTable 2 in Supplement 1). The meningioma-specific cumulative mortality was 1.2% (95% CI, 0.1%-2.2%), 3.0% (95% CI, 1.1%-5.0%), and 5.4% (95% CI, 2.4%-8.3%) at 5, 10, and 15 years from first meningioma diagnosis, respectively. The mortality rate per 1000 person years after subsequent neoplasm diagnosis for survivors who developed subsequent meningioma and those who developed other types of subsequent neoplasms was 4.89 (95% CI, 3.88-6.18) and 9.72 (95% CI, 9.06-10.40) (*P* < .001), respectively.

**Table 3.** Causes of Death Among Survivors With Subsequent Meningiomas

Characteristic	Patient, No. (%)				*P* value, comparing any vs no meningioma
	Any meningioma	≥2 Meningioma	Meningiomatosis^a^	No meningioma	
Cause of death					
Deaths, No.	71	13	4	3819	<.001
Recurrence of original cancer	4 (5.6)	0	0	1176 (31.2)	
Subsequent neoplasm	33 (46.5)	7 (53.8)	2 (50.0)	1009 (26.3)	
Nonmeningioma	8 (11.3)	0	0	1009 (26.3)	
Meningioma	25 (35.2)	7 (53.8)	2 (50.0)	0	
Cardiac causes	5 (7.0)	0	0	338 (8.6)	
Pulmonary causes	4 (5.6)	0	0	142 (3.8)	
External causes	5 (7.0)	2 (15.4)	1 (25.0)	335 (9.0)	
Other causes	16 (22.5)	3 (23.1)	1 (25.0)	534 (13.9)	
Unknown	4 (5.6)	1 (7.7)	0	285 (7.2)	

^a^ Meningiomatosis is defined by the ICD-0-3 diagnosis code 9530.1 or multiple meningiomas diagnosed the same date.

## Discussion

This cohort study examined the incidence, risk factors, and outcomes of childhood cancer survivors with subsequent meningiomas and was among the largest assembled cohort of this subsequent neoplasm. In addition to CRT exposure, young age at cancer diagnosis, and female sex, this study identified exposure to platinum chemotherapy, antimetabolite chemotherapy, and intrathecal methotrexate as risk factors for subsequent meningiomas. The study also found that non-Hispanic Black race was associated with a lower risk for subsequent meningioma. Furthermore, this study characterized a subgroup who are at risk of multiple meningiomas and meningiomatosis. Lastly, it provided further insights into late mortality among childhood cancer survivors with subsequent meningiomas. Compared with the prior CCSS report on meningiomas,^10^ this study included a substantially larger sample size (an additional 8101 survivors exposed to cranial radiation), longer follow-up (median of 35 years compared with a 42 years), and an examination of therapeutic risk factors for meningiomas and multiple meningiomas (whereas the 2017 article^10^ focused primarily upon the morbidity and mortality associated with meningiomas).

Exposure to platinum and antimetabolite chemotherapy have been inconsistently identified as risk factors for the development of subsequent meningiomas. For example, Kok et al’s study^8^ from the Dutch Childhood Oncology Group—Long-Term Effects After Childhood Cancer group identified exposure to carboplatin (HR, 3.55 [95% CI, 1.62-7.78]) being associated with increased meningioma risk. Furthermore, platinum-induced mutational signatures have been identified in secondary glioma, solid tumors, and therapy-related AML from childhood cancer survivors,^31,32^ with driver mutations predicted to be platinum-induced.^32^ Exposure to both intrathecal and systemic methotrexate have been reported as risk factors in some but not all studies.^7,12,33^ However, we provided evidence that exposure to both antimetabolite chemotherapy and intrathecal methotrexate as well as platinum agents were associated with a significantly increased risk for subsequent meningioma.

Exposure to radiation therapy and younger age at primary cancer diagnosis have long been recognized as risk factors for subsequent meningiomas. Indeed, among the cohort of 10 102 survivors who were not exposed to CRT, only 14 survivors (0.14%) reported a meningioma. Furthermore, there was an increased risk of meningiomas among female cancer survivors, consistent with the increased risk of de novo meningiomas observed in the general female population.^34^ Whether reduced ovarian function or hormone replacement therapies modify such risk for survivors should be evaluated in future studies. The identification of non-Hispanic Black race being associated with a decreased risk of subsequent meningioma has not been reported previously and contrasts with an increased risk of meningioma among Black individuals in the noncancer survivor population.^35,36^ It is possible that the decreased risk of meningioma among non-Hispanic Black survivors identified in this report reflects decreased access to and engagement with long-term follow-up care and screening.^37,38^

Although the overall survival of individuals with subsequent meningiomas is superior to other cancer survivors with subsequent malignant neoplasms, it is substantial and is similar to the 10-year survival of 94.2% (95% CI, 92.8%-95.4%) for people in the general population aged 20 to 44 years with meningiomas.^36^ Furthermore, this study highlighted a continued decline in long-term survival rates through 10 and 15 years after meningioma diagnosis without signs of slowing. The 15-year overall (18.4%) and subsequent meningioma-specific (5.4%) mortality rate reported in this study highlighted the substantial risk of death and complex morbidity and mortality risks of this population. These results provided justification and evidence for primary treating clinicians to consider when both screening and assessing the need for intervention vs cautious observation.

### Limitations

This study has limitations. First, although subsequent meningiomas were initially identified through self-report, they were confirmed by pathology reports or other medical records, and the true incidence of subsequent meningiomas may be underreported. Also, we do not know whether meningiomas were asymptomatic and detected on surveillance MRIs, which may result in higher rates of meningiomas compared with studies reporting on symptomatic meningiomas. Germline data were not available at time of analysis, thus the prevalence of genetic predisposition for meningioma risk among the cohort of survivors is unknown. Inherited predispositions to meningiomas, such as neurofibromatosis type 2, nevoid basal cell carcinoma syndrome, multiple endocrine neoplasia 1 (MEN1), Cowden syndrome, Werner syndrome, BAP1 tumor predisposition syndrome, Rubinstein-Taybi syndrome, and familial meningiomatosis caused by germline variants in the *SMARCB1* and *SMARCE1* genes, may impact the incidence of subsequent meningiomas, multiple meningiomas, and potentially the prognosis among childhood cancer survivors.^39^ Furthermore, biologic descriptions of the subsequent meningioma cases were limited to histologic coding. Recently, a molecular integrated grading scheme was proposed for meningioma prognostication,^40^ and future studies should investigate prognostic molecular risk factors in this population. Lastly, as with all longitudinal cohort studies, results may change as the cohort continues to age and more meningiomas are identified.

A central limitation of this study—and a longstanding challenge in the field of radiation-induced malignant neoplasms—is the difficulty in establishing clear correlations between radiation dose, irradiated volume, and the risk of subsequent neoplasms. Because few investigations provide detailed dosimetric reconstructions at the site of secondary tumor development, it remains uncertain whether these tumors arise within high-dose radiation fields or in regions exposed to lower doses. In the CCSS cohort, localization of the meningioma within the radiation field is not precise; thus, we are not able to draw direct correlations between dose of radiation to a location within the CNS and subsequent meningioma risk. However, we recognize that the tumor types may serve as proxies for radiation dose and whole brain vs focal radiation therapy. For example, survivors with a history of medulloblastoma were treated with craniospinal doses of 23.4 Gy to 36 Gy and a boost to 54 Gy to the posterior fossa (eg, high-dose cranial radiation); astrocytoma were treated with 45 Gy to 54 Gy to a region of the brain (eg, high-dose focal radiation); and leukemia were treated with 18 Gy to 24 Gy to the whole brain (eg, low-dose cranial radiation). Correspondingly, our analyses identified a cumulative incidence of subsequent meningiomas of 11.6% (95% CI, 9.0%-14.6%), 4.3% (95% CI, 2.5%-6.8%), and 3.6% (95% CI, 3.1%-4.1%), respectively, for survivors of medulloblastoma, astrocytoma, and leukemia.

## Conclusions

In this study of 24 886 childhood cancer survivors from the CCSS cohort, the risk of subsequent meningiomas remains across treatment eras, without a plateau across the lifespan. Younger diagnostic age, female sex, and exposure to CRT, platinum agents, antimetabolites, and intrathecal methotrexate were identified as independent meningioma risk factors. The detailed cumulative incidence by primary cancer diagnosis as well as risk factors identified could be used for future incorporation into survivorship guidelines. The risk for meningioma-associated mortality, combined with prior reports of associated morbidity, argues for assertive meningioma surveillance and treatment in this population.

## Notes

Supplement 1. **eTable 1.** Cumulative Incidence of First Meningioma at Different Years After Cancer Diagnosis **eTable 2.** Cumulative Incidence (%) of Death for Meningioma Survivors

Supplement 2.
Data Sharing Statement

## References

[1] Ehrhardt MJ, Krull KR, Bhakta N, . Improving quality and quantity of life for childhood cancer survivors globally in the twenty-first century. Nat Rev Clin Oncol. 2023;20(10):678-696. doi:10.1038/s41571-023-00802-w 37488230

[2] Dixon SB, Liu Q, Chow EJ, . Specific causes of excess late mortality and association with modifiable risk factors among survivors of childhood cancer: a report from the Childhood Cancer Survivor Study cohort. Lancet. 2023;401(10386):1447-1457. doi:10.1016/S0140-6736(22)02471-0 37030315 PMC10149583

[3] Gibson TM, Mostoufi-Moab S, Stratton KL, . Temporal patterns in the risk of chronic health conditions in survivors of childhood cancer diagnosed 1970-99: a report from the Childhood Cancer Survivor Study cohort. Lancet Oncol. 2018;19(12):1590-1601. doi:10.1016/S1470-2045(18)30537-0 30416076 PMC6309183

[4] Walter AW, Hancock ML, Pui CH, . Secondary brain tumors in children treated for acute lymphoblastic leukemia at St Jude Children’s Research Hospital. J Clin Oncol. 1998;16(12):3761-3767. doi:10.1200/JCO.1998.16.12.3761 9850019

[5] Neglia JP, Robison LL, Stovall M, . New primary neoplasms of the central nervous system in survivors of childhood cancer: a report from the Childhood Cancer Survivor Study. J Natl Cancer Inst. 2006;98(21):1528-1537. doi:10.1093/jnci/djj411 17077355

[6] Rimm IJ, Li FC, Tarbell NJ, Winston KR, Sallan SE. Brain tumors after cranial irradiation for childhood acute lymphoblastic leukemia: a 13-year experience from the Dana-Farber Cancer Institute and the Children’s Hospital. Cancer. 1987;59(8):1506-1508. doi:10.1002/1097-0142(19870415)59:8<1506::AID-CNCR2820590819>3.0.CO;2-P 3545441

[7] Withrow DR, Anderson H, Armstrong GT, . Pooled analysis of meningioma risk following treatment for childhood cancer. JAMA Oncol. 2022;8(12):1756-1764. doi:10.1001/jamaoncol.2022.4425 36201196 PMC9539736

[8] Kok JL, Teepen JC, van Leeuwen FE, ; DCOG-LATER Study Group. Risk of benign meningioma after childhood cancer in the DCOG-LATER cohort: contributions of radiation dose, exposed cranial volume, and age. Neuro Oncol. 2019;21(3):392-403. doi:10.1093/neuonc/noy124 30099534 PMC6380414

[9] Friedman DL, Whitton J, Leisenring W, . Subsequent neoplasms in 5-year survivors of childhood cancer: the Childhood Cancer Survivor Study. J Natl Cancer Inst. 2010;102(14):1083-1095. doi:10.1093/jnci/djq238 20634481 PMC2907408

[10] Bowers DC, Moskowitz CS, Chou JF, . Morbidity and mortality associated with meningioma after cranial radiotherapy: a report from the childhood cancer survivor study. J Clin Oncol. 2017;35(14):1570-1576. doi:10.1200/JCO.2016.70.1896 28339329 PMC5455703

[11] Verbruggen LC, Hudson MM, Bowers DC, . Variations in screening and management practices for subsequent asymptomatic meningiomas in childhood, adolescent and young adult cancer survivors. J Neurooncol. 2020;147(2):417-425. doi:10.1007/s11060-020-03436-5 32088813

[12] Taylor AJ, Little MP, Winter DL, . Population-based risks of CNS tumors in survivors of childhood cancer: the British Childhood Cancer Survivor Study. J Clin Oncol. 2010;28(36):5287-5293. doi:10.1200/JCO.2009.27.0090 21079138 PMC4809645

[13] Gold DG, Neglia JP, Dusenbery KE. Second neoplasms after megavoltage radiation for pediatric tumors. Cancer. 2003;97(10):2588-2596. doi:10.1002/cncr.11356 12733158

[14] Galloway TJ, Indelicato DJ, Amdur RJ, Swanson EL, Smith AA, Marcus RB Jr. Second tumors in pediatric patients treated with radiotherapy to the central nervous system. Am J Clin Oncol. 2012;35(3):279-283. doi:10.1097/COC.0b013e318210f533 21383606

[15] Chojnacka M, Pędziwiatr K, Skowrońska-Gardas A, Perek-Polnik M, Perek D, Olasek P. Second brain tumors following central nervous system radiotherapy in childhood. Br J Radiol. 2014;87(1041):20140211. doi:10.1259/bjr.20140211 24968876 PMC4453152

[16] Bowers DC, Nathan PC, Constine L, . Subsequent neoplasms of the CNS among survivors of childhood cancer: a systematic review. Lancet Oncol. 2013;14(8):e321-e328. doi:10.1016/S1470-2045(13)70107-4 23816298 PMC4522926

[17] Goshen Y, Stark B, Kornreich L, Michowiz S, Feinmesser M, Yaniv I. High incidence of meningioma in cranial irradiated survivors of childhood acute lymphoblastic leukemia. Pediatr Blood Cancer. 2007;49(3):294-297. doi:10.1002/pbc.21153 17243137

[18] Kutsenko A, Berrington de Gonzalez A, Curtis RE, Rajaraman P. Risk of second benign brain tumors among cancer survivors in the surveillance, epidemiology, and end results program. Cancer Causes Control. 2014;25(6):659-668. doi:10.1007/s10552-014-0367-5 24682745

[19] Casey DL, Vogelius IR, Brodin NP, . Risk of subsequent neoplasms in childhood cancer survivors after radiation therapy: a PENTEC comprehensive review. Int J Radiat Oncol Biol Phys. 2024;119(2):640-654. doi:10.1016/j.ijrobp.2023.07.025 37777927

[20] Taylor AJ, Frobisher C, Ellison DW, . Survival after second primary neoplasms of the brain or spinal cord in survivors of childhood cancer: results from the British Childhood Cancer Survivor Study. J Clin Oncol. 2009;27(34):5781-5787. doi:10.1200/JCO.2009.22.4386 19786666

[21] Armstrong GT, Liu Q, Yasui Y, . Long-term outcomes among adult survivors of childhood central nervous system malignancies in the Childhood Cancer Survivor Study. J Natl Cancer Inst. 2009;101(13):946-958. doi:10.1093/jnci/djp148 19535780 PMC2704230

[22] Robison LL, Mertens AC, Boice JD, . Study design and cohort characteristics of the Childhood Cancer Survivor Study: a multi-institutional collaborative project. Med Pediatr Oncol. 2002;38(4):229-239. doi:10.1002/mpo.1316 11920786

[23] Robison LL, Armstrong GT, Boice JD, . The Childhood Cancer Survivor Study: a National Cancer Institute-supported resource for outcome and intervention research. J Clin Oncol. 2009;27(14):2308-2318. doi:10.1200/JCO.2009.22.3339 19364948 PMC2677920

[24] Packer RJ, Gurney JG, Punyko JA, . Long-term neurologic and neurosensory sequelae in adult survivors of a childhood brain tumor: childhood cancer survivor study. J Clin Oncol. 2003;21(17):3255-3261. doi:10.1200/JCO.2003.01.202 12947060

[25] Howell RM, Smith SA, Weathers RE, Kry SF, Stovall M. Adaptations to a generalized radiation dose reconstruction methodology for use in epidemiologic studies: an update from the MD Anderson late effect group. Radiat Res. 2019;192(2):169-188. doi:10.1667/RR15201.1 31211642 PMC8041091

[26] World Health Organization. International Classification of Diseases, Oncology*. *World Health Organization; 2018.

[27] *STATA Survival Analysis and Epidemiological Tables Reference Manual*. STATA Press; 2011.

[28] Zhang Z. Survival analysis in the presence of competing risks. Ann Transl Med. 2017;5(3):47. doi:10.21037/atm.2016.08.62 28251126 PMC5326634

[29] Green DM, Nolan VG, Goodman PJ, . The cyclophosphamide equivalent dose as an approach for quantifying alkylating agent exposure: a report from the Childhood Cancer Survivor Study. Pediatr Blood Cancer. 2014;61(1):53-67. doi:10.1002/pbc.24679 23940101 PMC3933293

[30] *SAS 9.4 Global Statements*. SAS Institute; 2017.

[31] Sánchez-Guixé M, Muiños F, Pinheiro-Santin M, . Origins of second malignancies in children and mutational footprint of chemotherapy in normal tissues. Cancer Discov. 2024;14(6):953-964. doi:10.1158/2159-8290.CD-23-1186 38501975 PMC11145171

[32] Schwartz JR, Ma J, Kamens J, . The acquisition of molecular drivers in pediatric therapy-related myeloid neoplasms. Nat Commun. 2021;12(1):985. doi:10.1038/s41467-021-21255-8 33579957 PMC7880998

[33] Journy NMY, Zrafi WS, Bolle S, . Risk Factors of subsequent central nervous system tumors after childhood and adolescent cancers: findings from the French Childhood Cancer Survivor Study. Cancer Epidemiol Biomarkers Prev. 2021;30(1):133-141. doi:10.1158/1055-9965.EPI-20-0735 33033142

[34] CBTRUS statistical report: primary brain and central nervous cystem tumors diagnosed in the United States in 2004-2008. Central Brain Tumor Registry of the United States. Accessed November 5, 2025. http://www.cbtrus.org

[35] Walsh KM, Price M, Neff C, . The joint impacts of sex and race/ethnicity on incidence of grade 1 versus grades 2-3 meningioma across the lifespan. Neurooncol Adv. 2023;5(suppl 1):i5-i12. doi:10.1093/noajnl/vdad020 37287573 PMC10243865

[36] Ostrom QT, Gittleman H, Liao P, . CBTRUS statistical report: primary brain and other central nervous system tumors diagnosed in the United States in 2010-2014. Neuro Oncol. 2017;19(suppl 5):v1-v88. doi:10.1093/neuonc/nox158 29117289 PMC5693142

[37] Klosky JL, Cash DK, Buscemi J, . Factors influencing long-term follow-up clinic attendance among survivors of childhood cancer. J Cancer Surviv. 2008;2(4):225-232. doi:10.1007/s11764-008-0063-0 18787958 PMC2652131

[38] Daly A, Lewis RW, Vangile K, . Survivor clinic attendance among pediatric- and adolescent-aged survivors of childhood cancer. J Cancer Surviv. 2019;13(1):56-65. doi:10.1007/s11764-018-0727-3 30560348

[39] Kerr K, Qualmann K, Esquenazi Y, Hagan J, Kim DH. Familial syndromes involving meningiomas provide mechanistic insight into sporadic disease. Neurosurgery. 2018;83(6):1107-1118. doi:10.1093/neuros/nyy121 29660026 PMC6235681

[40] Driver J, Hoffman SE, Tavakol S, . A molecularly integrated grade for meningioma. Neuro Oncol. 2022;24(5):796-808. doi:10.1093/neuonc/noab213 34508644 PMC9071299

