# Errors in Key Points, Abstract, Results, and Discussion

**DOI:** 10.1001/jamanetworkopen.2026.2085

**Published:** 2026-02-17

In the Original Investigation titled “Subsequent Meningiomas Among Survivors of Childhood Cancer,”^1^ published December 11, 2025, there were errors in the Key Points, Abstract, Results, and Discussion, in which it was incorrectly reported that 6-mercaptopurine (6-MP) was the antimetabolite exposure. However, in all analyses, the antimetabolite exposure was actually modeled as a composite class variable that included methotrexate, 6-MP, 6-thioguanine, and cytarabin. The language throughout the article has been updated to *antimetabolite chemotherapy*. The authors have explained the error in a Comment.^2^ This article has been corrected.^1^

## References

[1] Bowers DC, Cooney T, Chen Y, . Subsequent meningiomas among survivors of childhood cancer. JAMA Netw Open. 2025;8(12):e2548715. doi:10.1001/jamanetworkopen.2025.48715 41379444 PMC12699355

[zcx260007r2] 2. Bowers D. Author response to Acar/Correction. *JAMA Network Open*. Published December 31, 2025. Accessed January 23, 2026. https://jamanetwork.com/journals/jamanetworkopen/fullarticle/2842656

