# Supplementary material for: Subsequent Meningiomas Among Survivors of Childhood Cancer
Source: JAMA Netw Open. 2025 Dec 11;8(12):e2548715. doi: 10.1001/jamanetworkopen.2025.48715 (PMC12699355; doi:10.1001/jamanetworkopen.2025.48715)
Supplement: Supplement 1. — eTable 1. Cumulative Incidence of First Meningioma at Different Years After Cancer Diagnosis eTable 2. Cumulative Incidence (%) of Death for Meningioma Survivors [file jamanetwopen-e2548715-s001.pdf]

Supplemental Online Content

Bowers DC, Cooney T, Chen Y, et al. Subsequent meningiomas among survivors of childhood cancer. *JAMA Netw Open*. 2025;8(12):e2548715. doi:10.1001/jamanetworkopen.2025.48715

eTable 1. Cumulative incidence of first meningioma at different years after cancer diagnosis

eTable 2. Cumulative incidence (%) of death for meningioma survivors

This supplemental material has been provided by the authors to give readers additional information about their work.

© 2025 Bowers DC et al. *JAMA Network Open*.

eTable 1. Cumulative incidence of first meningioma at different years after cancer diagnosis

Characteristics		20	25	30	35
ALL	all	0.44 (0.37-0.53)	0.83 (0.73-0.95)	1.55 (1.39-1.73)	2.32 (2.09-2.56)
Sex	Female	0.44 (0.34-0.57)	0.88 (0.72-1.06)	1.90 (1.64-2.19)	2.93 (2.56-3.33)
	Male	0.44 (0.35-0.56)	0.79 (0.66-0.95)	1.25 (1.06-1.46)	1.79 (1.53-2.08)
Age at diagnosis	0-4	0.40 (0.30-0.52)	0.80 (0.65-0.98)	1.66 (1.41-1.95)	2.58 (2.22-2.99)
	5-9	0.58 (0.42-0.78)	1.07 (0.83-1.35)	1.82 (1.48-2.22)	2.80 (2.31-3.37)
	10-14	0.47 (0.32-0.69)	0.91 (0.67-1.21)	1.60 (1.24-2.02)	2.17 (1.72-2.71)
	15-20	0.31 (0.17-0.53)	0.43 (0.26-0.69)	0.72 (0.47-1.06)	1.02 (0.69-1.46)
Cancer diagnosis	Acute lymphoblastic leukemia	0.44 (0.32-0.58)	1.00 (0.81-1.22)	2.24 (1.91-2.61)	3.59 (3.10-4.12)
	Acute myeloid leukemia	0.00 (0.00-0.00)	0.33 (0.07-1.13)	0.98 (0.37-2.19)	2.24 (1.07-4.15)
	Astrocytoma	0.61 (0.36-0.96)	1.35 (0.94-1.89)	2.41 (1.78-3.18)	3.55 (2.65-4.64)
	Ewings sarcoma	0.14 (0.01-0.75)	0.14 (0.01-0.75)	0.14 (0.01-0.75)	0.47 (0.08-1.75)
	Hodgkin lymphoma	0.00 (0.00-0.00)	0.07 (0.02-0.26)	0.17 (0.06-0.43)	0.35 (0.15-0.70)
	Kidney tumors	0.00 (0.00-0.00)	0.06 (0.01-0.32)	0.12 (0.03-0.41)	0.12 (0.03-0.41)
	Medulloblastoma	4.08 (2.99-5.41)	5.65 (4.29-7.26)	8.63 (6.74-10.81)	11.61 (9.01-14.55)
	Neuroblastoma	0.16 (0.05-0.44)	0.28 (0.11-0.64)	0.43 (0.19-0.87)	0.66 (0.32-1.26)
	Non-Hodgkin lymphoma	0.25 (0.10-0.56)	0.42 (0.20-0.81)	0.49 (0.25-0.92)	1.33 (0.76-2.19)
	Osteosarcoma	0.08 (0.01-0.44)	0.08 (0.01-0.44)	0.08 (0.01-0.44)	0.08 (0.01-0.44)
	Other CNS tumors	1.50 (0.77-2.67)	1.72 (0.91-2.98)	3.79 (2.18-6.09)	4.31 (2.49-6.87)
	Other leukemia	0.00 (0.00-0.00)	0.67 (0.14-2.25)	1.26 (0.33-3.52)	2.04 (0.62-5.07)
	Soft tissue sarcoma	0.25 (0.07-0.69)	0.52 (0.22-1.10)	0.89 (0.44-1.65)	1.08 (0.54-1.97)
Decade of diagnosis of original cancer	1970-1979	0.58 (0.41-0.79)	1.11 (0.87-1.39)	1.96 (1.64-2.33)	2.86 (2.47-3.30)
	1980-1989	0.39 (0.28-0.52)	0.76 (0.61-0.94)	1.30 (1.09-1.53)	1.87 (1.59-2.18)
	1990-1999	0.42 (0.32-0.55)	0.74 (0.56-0.95)	1.42 (1.05-1.88)	1.42 (1.05-1.88)
CRT dose	>0->=30	0.45 (0.34-0.60)	0.96 (0.78-1.18)	1.90 (1.62-2.21)	2.92 (2.56-3.33)
	>30->=50	1.46 (0.84-2.38)	3.43 (2.38-4.76)	5.78 (4.34-7.51)	7.13 (5.46-9.09)
	>50Gy	2.73 (2.04-3.58)	3.97 (3.09-5.01)	6.10 (4.89-7.50)	9.01 (7.18-11.09)
	None	0.04 (0.02-0.09)	0.08 (0.04-0.14)	0.14 (0.08-0.25)	0.14 (0.08-0.25)

© 2025 Bowers DC et al. *JAMA Network Open*.

eTable 2. Cumulative incidence (%) of death for meningioma survivors

			Year after meningioma diagnosis					
**Cause of death**	**Diagnosis Era**	**# of deaths**	**1**	**2**	**3**	**5**	**10**	**15**
Any	Any	71	1.44 (0.38-2.50)	2.11 (0.81-3.40)	3.57 (1.84-5.29)	4.91 (2.85-6.97)	10.50 (7.11-13.88)	18.37 (13.32-23.42)
	1970-1979	49	0.43 (0.00-1.28)	1.31 (0.00-2.79)	3.22 (0.87-5.57)	4.70 (1.85-7.55)	10.79 (6.25-15.33)	20.80 (14.07-27.53)
	1980-1989	17	1.76 (0.00-3.74)	2.38 (0.07-4.69)	3.69 (0.79-6.59)	5.29 (1.68-8.89)	8.43 (3.46-13.40)	11.93 (5.18-18.68)
	1990-1999	5	3.77 (0.00-7.95)	3.77 (0.00-7.95)	3.77 (0.00-7.95)	3.77 (0.00-7.95)	14.55 (0.00-29.12)	14.55 (0.00-29.12)
Subsequent meningioma	Any	25	0.21 (0.00-0.61)	0.65 (0.00-1.39)	0.89 (0.02-1.76)	1.16 (0.14-2.18)	3.04 (1.11-4.96)	5.37 (2.41-8.33)
	1970-1979	15	0.00 (0.00-0.00)	0.44 (0.00-1.31)	0.44 (0.00-1.31)	0.44 (0.00-1.31)	2.31 (0.05-4.57)	4.97 (1.26-8.67)
	1980-1989	8	0.00 (0.00-0.00)	0.62 (0.00-1.83)	1.27 (0.00-3.02)	2.04 (0.00-4.35)	2.90 (0.07-5.72)	4.56 (0.30-8.82)
	1990-1999	2	1.27 (0.00-3.74)	1.27 (0.00-3.74)	1.27 (0.00-3.74)	1.27 (0.00-3.74)	6.66 (0.00-17.19)	6.66 (0.00-17.19)

© 2025 Bowers DC et al. *JAMA Network Open*.
