# Supplementary material for: Subsequent Meningiomas Among Survivors of Childhood Cancer
Source: JAMA Netw Open. 2025 Dec 11;8(12):e2548715. doi: 10.1001/jamanetworkopen.2025.48715 (PMC12699355; doi:10.1001/jamanetworkopen.2025.48715)
Supplement: Supplement 2. — Data Sharing Statement [file jamanetwopen-e2548715-s002.pdf]

Data Sharing Statement

Data
Data available: Yes
Data types: Deidentified participant data
How to access data: https://ccss.stjude.org/public-access-data.html
When available: With publication

Supporting Documents
Document types: Other (please specify)
Additional Information: self-reported data
How to access documents: self-reported data
When available: With publication

Additional Information
Who can access the data: Anyone requesting the data
Types of analyses: For any purpose.
Mechanisms of data availability: Raw data is available for review.
Any additional restrictions: None.
